# Supplementary material for: PTEN restrains SHH medulloblastma growth through cell autonomous and nonautonomous mechanisms
Source: bioRxiv. 2025 Aug 2:2025.07.31.667996. Preprint. [Version 1] doi: 10.1101/2025.07.31.667996 (PMC12324473; doi:10.1101/2025.07.31.667996)
Supplement: 1 [file NIHPP2025.07.31.667996V1-supplement-1.pdf]

bioRxiv preprint doi: https://doi.org/10.1101/2025.07.31.667996; this version posted August 2, 2025. The copyright holder for this preprint (which was not certified by peer review) is the author/funder, who has granted bioRxiv a license to display the preprint in perpetuity. It is made available under aCC-BY 4.0 International license.

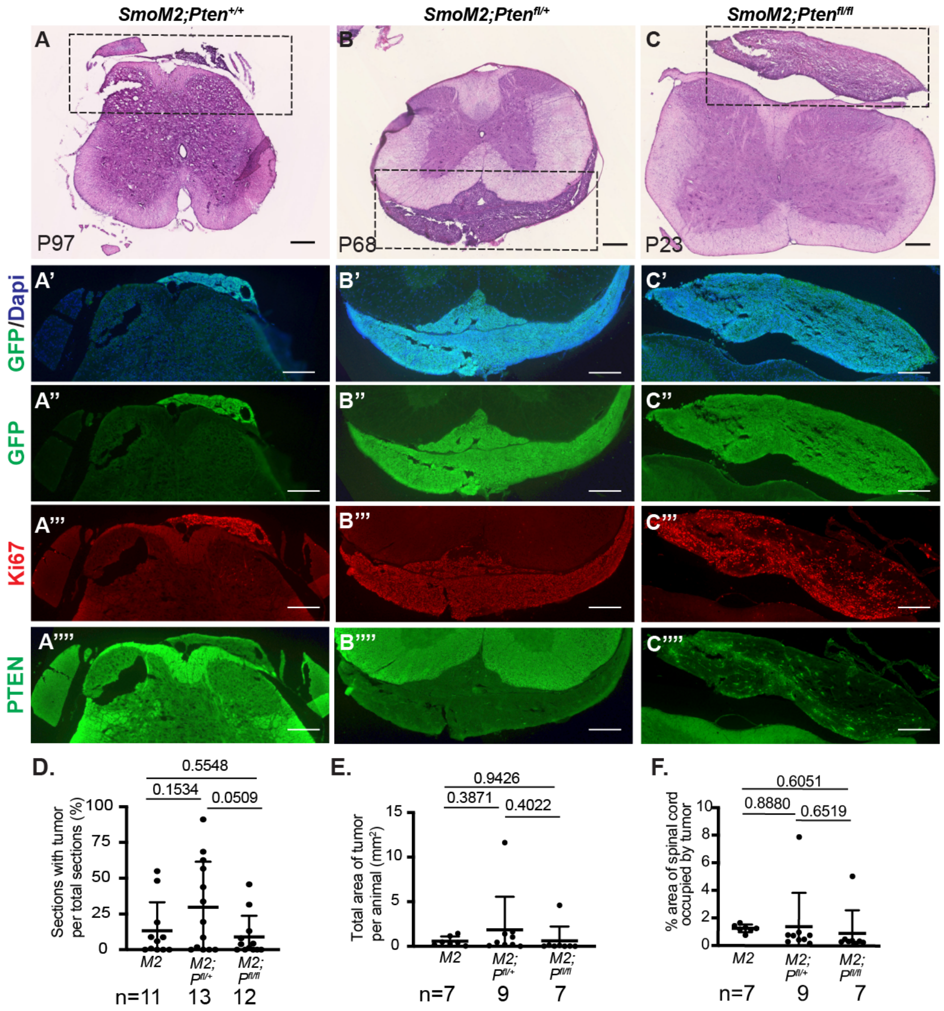

Figure 1–Figure Supplement 1. Loss of *Pten* does not increase metastasis to the spinal cord in *SmoM2* SHH-MB

(**A-C**) Coronal sections of spinal cords stained with H&E from mice of the indicated three genotypes showing metastatic lesions at end stage. Dashed rectangles indicate locations of images below stained for the proteins and DAPI indicated on the left. Scale bars indicates 200μm. (**D**) Quantification of percentage of sections with a tumor for each animal analyzed. (**E**) Quantification of the total area of the tumor on all the sections. (**F**) Quantification of the area of each tumor as a percentage of the spinal cord area in each section. (D-F) Number of animals per genotype (n=) are indicated below graphs. All statistics were determined using an unpaired t-test comparing *SmoM2* tumors to each of the *Pten* mutants or the *Pten* mutants to each other.

bioRxiv preprint doi: https://doi.org/10.1101/2025.07.31.667996; this version posted August 2, 2025. The copyright holder for this preprint (which was not certified by peer review) is the author/funder, who has granted bioRxiv a license to display the preprint in perpetuity. It is made available under aCC-BY 4.0 International license.

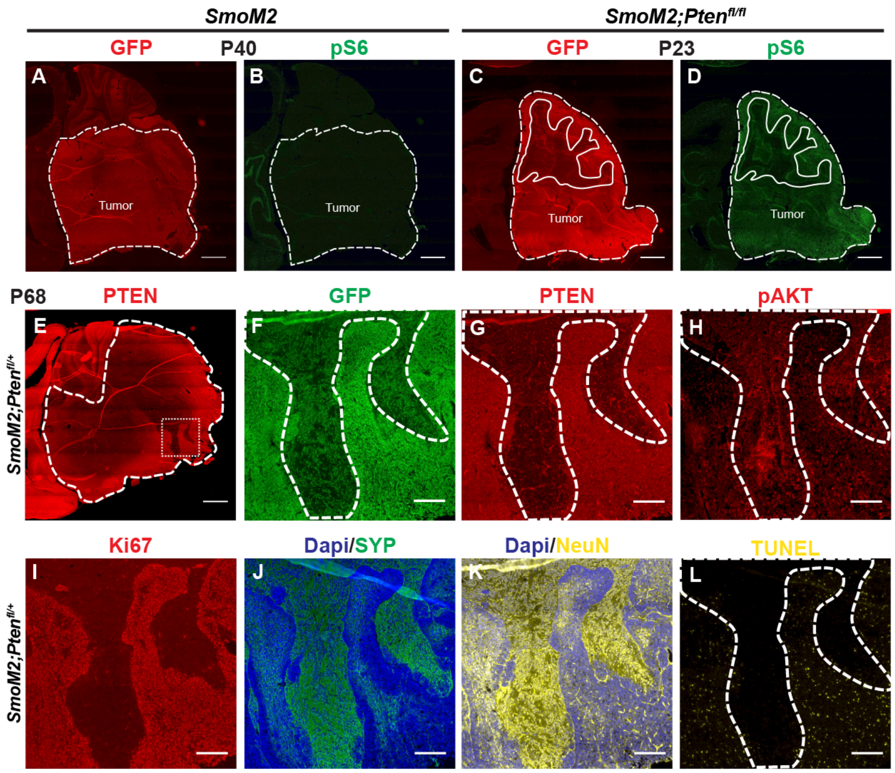

Figure 2–Figure Supplement 1. Spontaneous loss of the second allele of *Pten* in rare cells of some *SmoM2-Pten*$^{fl/+}$ tumors

(**A-D**) Sagittal sections of end stage tumors (GFP+) showing upregulation of pS6 in *SmoM2-Pten*$^{fl/fl}$ mice compared to *SmoM2* (n=3 per genotype). Dashed lines outline the tumors, solid lines outline IGL. Scale bars indicates 1mm. (**E**) Section of end stage tumor from *SmoM2-Pten*$^{fl/+}$ mice showing coherent patches of tumor cells (GFP+) devoid of PTEN (n=3). Dashed line square indicates location of high magnification images shown in (F-L). Scale bar indicates 1mm. (**F-L**) Sections of lateral tumors stained for the indicated protein and DAPI (n=3). Dashed line outlines tumor area where PTEN is absent (G) or pAKT (H), SYP (J) and NeuN (K) are upregulated and proliferation (Ki67) and cell death (TUNEL) are reduced (I, L). Scale bars indicates 200μm.

bioRxiv preprint doi: https://doi.org/10.1101/2025.07.31.667996; this version posted August 2, 2025. The copyright holder for this preprint (which was not certified by peer review) is the author/funder, who has granted bioRxiv a license to display the preprint in perpetuity. It is made available under aCC-BY 4.0 International license.

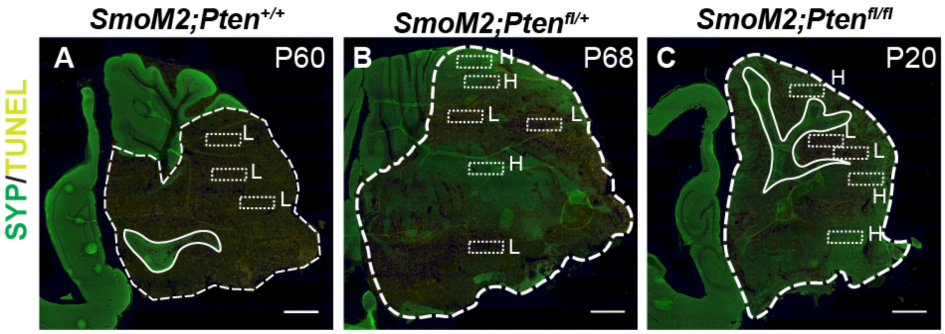

Figure 3–figure supplement 1. Method of quantifying TUNEL particles in tumors
(**A-C**) Examples of stained sagittal cerebellar sections showing where quantifications of density of TUNEL particles were performed in the three genotypes in end stage lateral tumor regions distinguishing high SYP (H) compared to low SYP (L) regions. Dashed lines outline each tumor, solid lines outline IGL, dotted boxes indicate high SYP (H) and low SYP (L) regions. Scale bars indicate 1mm.

bioRxiv preprint doi: https://doi.org/10.1101/2025.07.31.667996; this version posted August 2, 2025. The copyright holder for this preprint (which was not certified by peer review) is the author/funder, who has granted bioRxiv a license to display the preprint in perpetuity. It is made available under aCC-BY 4.0 International license.

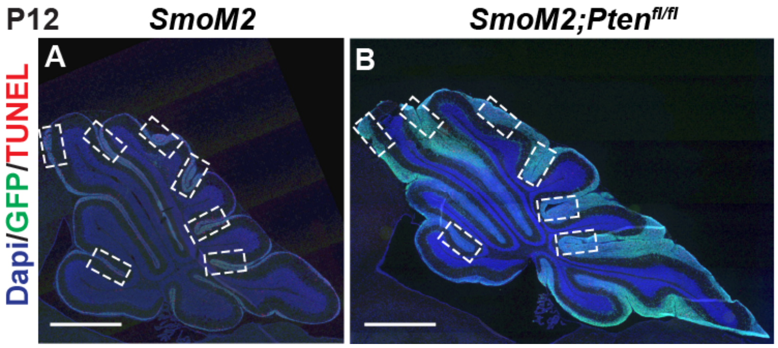

Figure 4–figure supplement 1. Method of quantifying TUNEL particles in P12 EGL
(**A, B**) Examples of stained midline sagittal cerebellar sections showing where quantification of density of TUNEL particles was performed in the EGL in medial regions of the cerebellum. Scale bars indicate 1mm.

bioRxiv preprint doi: https://doi.org/10.1101/2025.07.31.667996; this version posted August 2, 2025. The copyright holder for this preprint (which was not certified by peer review) is the author/funder, who has granted bioRxiv a license to display the preprint in perpetuity. It is made available under aCC-BY 4.0 International license.

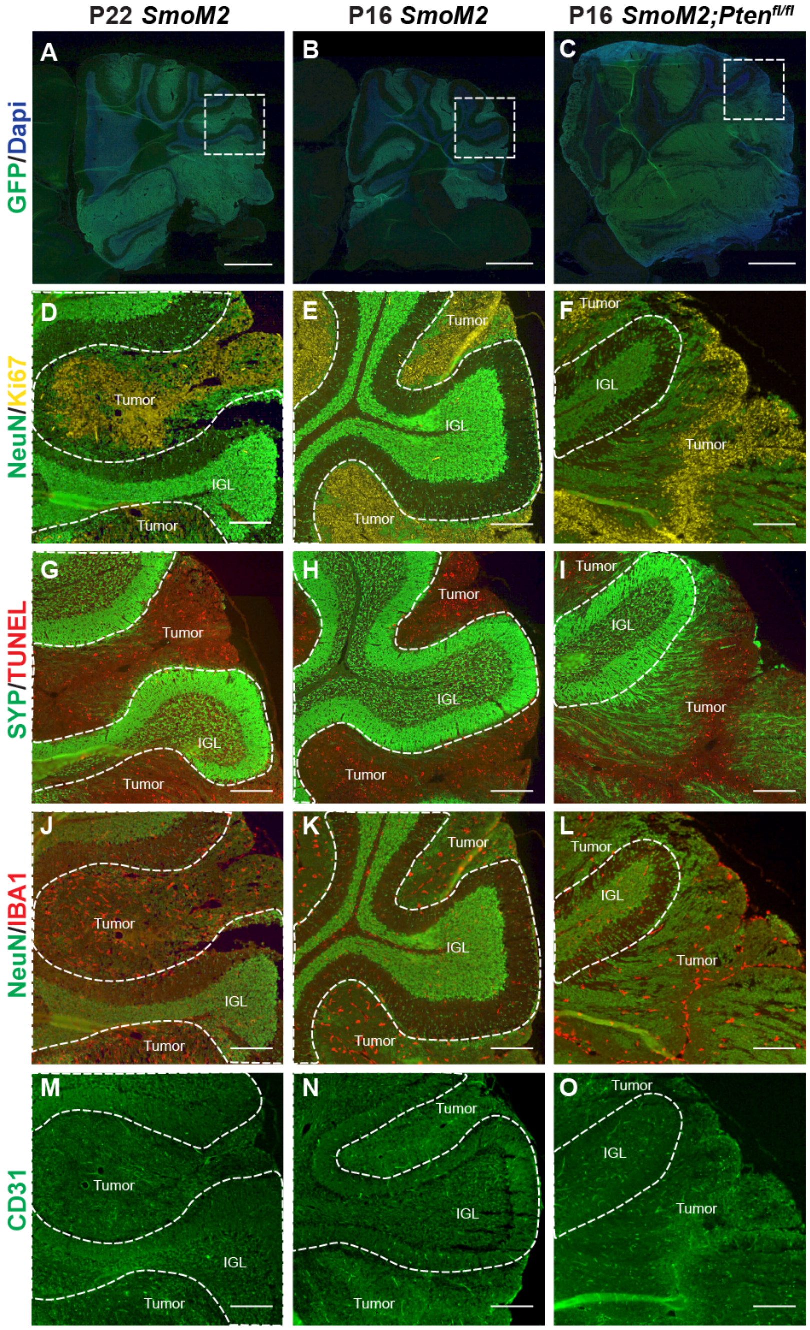

Figure 8–figure supplement 1 The cellular phenotypes of large tumors from P16 *SmoM2-Pten*$^{fl/fl}$ mice and P22 *SmoM2* mice are similar to end stage

(**A-C**) Sagittal sections of cerebellar sections from P16 and P22 *SmoM2* mice and P16 *SmoM2-Pten*$^{fl/fl}$ mice stained with GFP and DAPI to highlight tumor cells (n=3 mice/genotype). Dashed line squares indicate location of high magnification images shown in (D-O). Scale bars indicate 1mm. (**D-O**) Higher magnification images of tumors stained for indicated proteins to confirm that the cellular phenotypes of large tumors in P16 *SmoM2-Pten*$^{fl/fl}$ mice and P22 *SmoM2* mice are like tumors at end stage. Dashed lines outline the internal granule layer (IGL). Scale bars indicate 200μm.

bioRxiv preprint doi: https://doi.org/10.1101/2025.07.31.667996; this version posted August 2, 2025. The copyright holder for this preprint (which was not certified by peer review) is the author/funder, who has granted bioRxiv a license to display the preprint in perpetuity. It is made available under aCC-BY 4.0 International license.

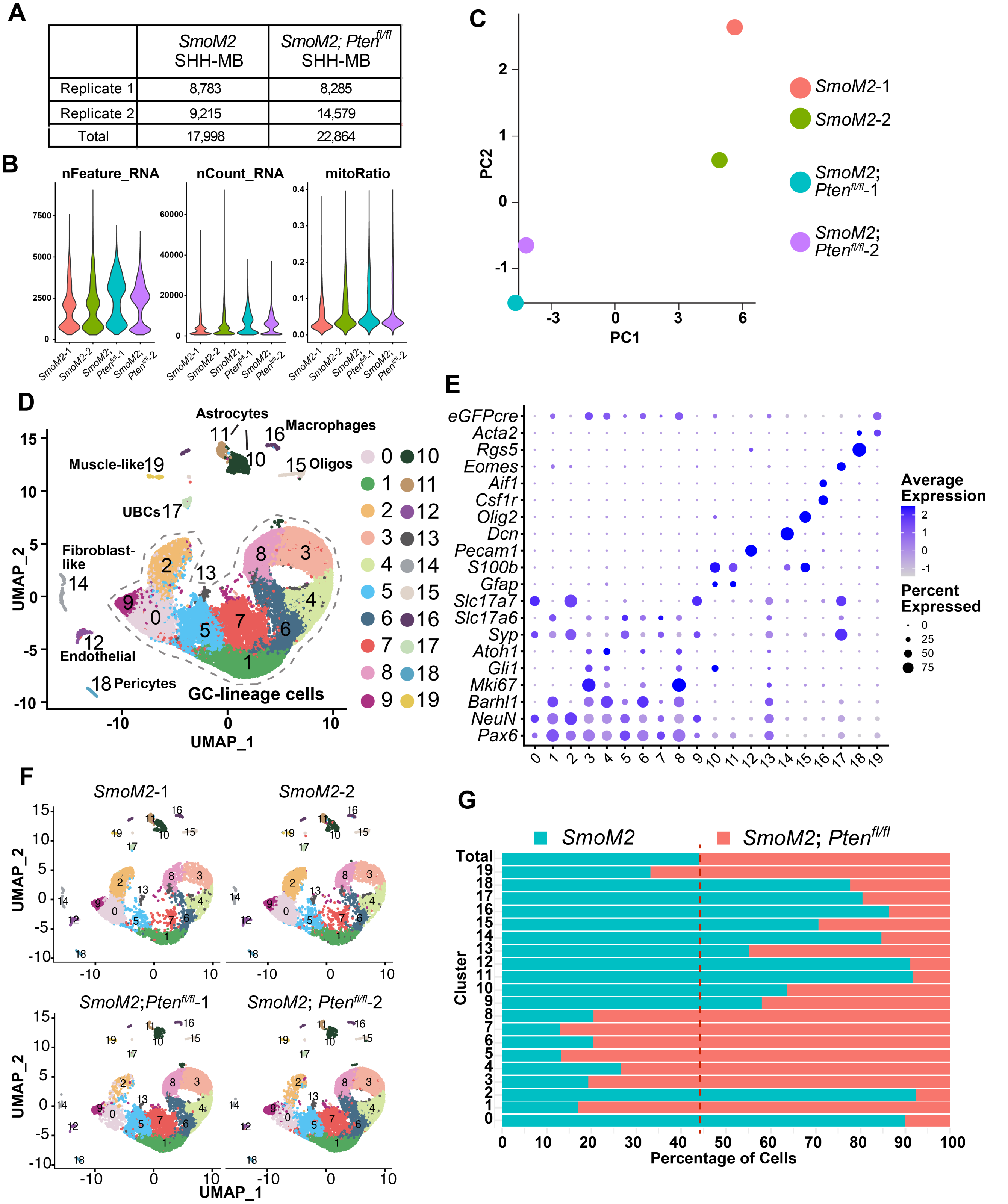

Figure 8–figure supplement 2. ScRNA-seq comparison of tumors from P16 *SmoM2-Pten$^{fl/fl}$* mice to P22 *SmoM2* mice

bioRxiv preprint doi: https://doi.org/10.1101/2025.07.31.667996; this version posted August 2, 2025. The copyright holder for this preprint (which was not certified by peer review) is the author/funder, who has granted bioRxiv a license to display the preprint in perpetuity. It is made available under aCC-BY 4.0 International license.

(**A**) Number of cells from each replicate and genotype used for downstream analyses after filtering. (**B**) Violin plots showing the number of features, and RNA counts and percent mitochondrial RNA counts across the biological replicates of the scRNA-seq data set after filtering out poor quality cells (where number of detected genes was ≤ 500, the number of detected transcripts was ≥ 18,000 and mitochondrial gene percentage ≥ 20%). (**C**) PCA plot after filtering out poor quality cells and integrating the replicates and genotypes showing the two genotypes separate well. (**D**) UMAP showing projections of all cells showing cluster annotations. (**E**) Dot plot graph showing expression levels of cell type marker genes across all clusters and their cell type desgnations. (**F**) UMAPs of all cells shown by genotype and sample. (**G**) Graph showing proportions of cells of each genotype when all cells are included. Dotted line represents the expected ratio between the two genotypes.

bioRxiv preprint doi: https://doi.org/10.1101/2025.07.31.667996; this version posted August 2, 2025. The copyright holder for this preprint (which was not certified by peer review) is the author/funder, who has granted bioRxiv a license to display the preprint in perpetuity. It is made available under aCC-BY 4.0 International license.

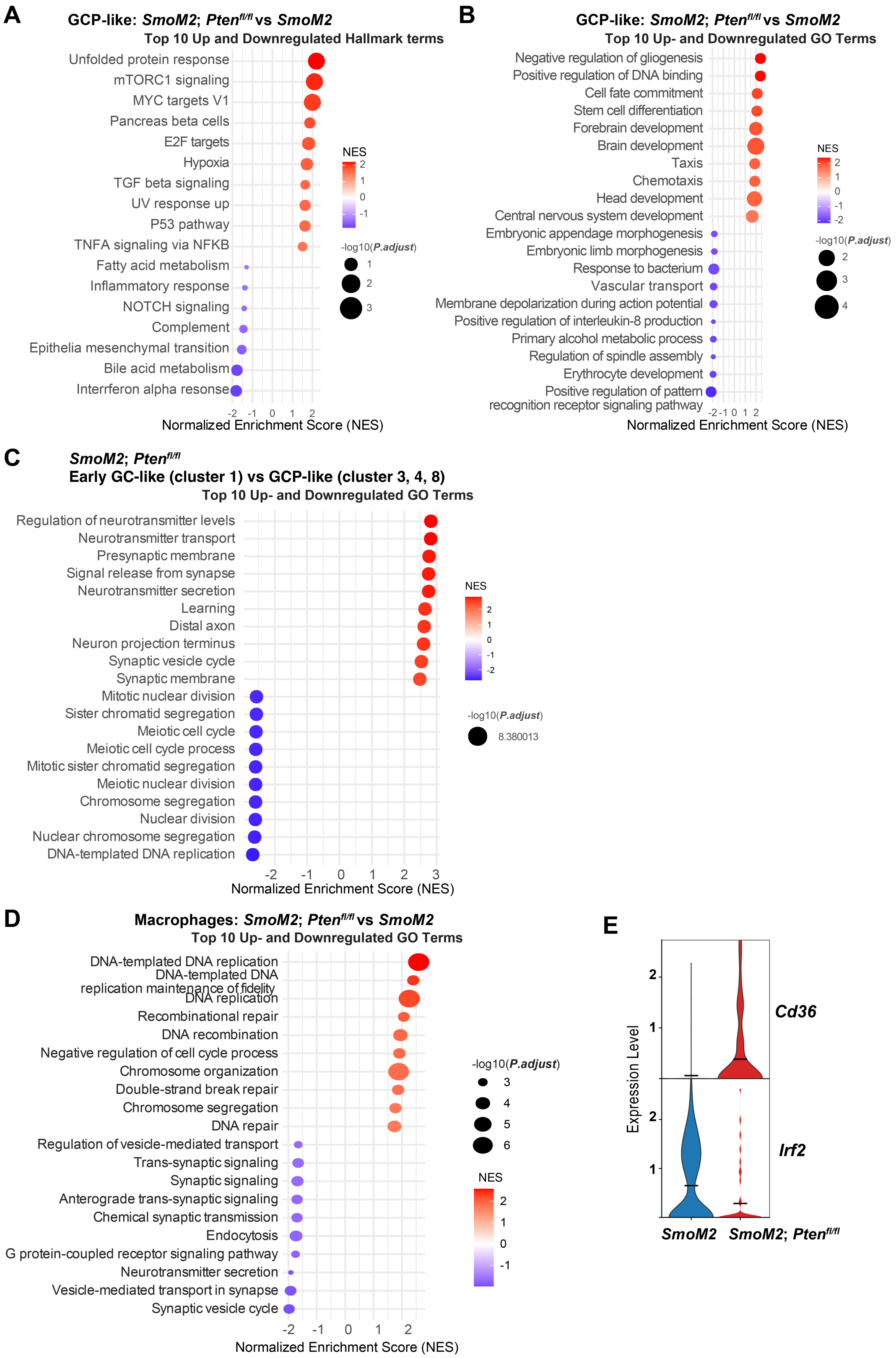

Figure 9–figure supplement 1. Differential expression analyses confirms activation of mTOR signaling, altered neural differentiation and decreased immune signaling following *Pten* loss

bioRxiv preprint doi: https://doi.org/10.1101/2025.07.31.667996; this version posted August 2, 2025. The copyright holder for this preprint (which was not certified by peer review) is the author/funder, who has granted bioRxiv a license to display the preprint in perpetuity. It is made available under aCC-BY 4.0 International license.

A. Bar plots showing top 10 upregulated and downregulated Hallmark terms in *SmoM2-Pten$^{fl/fl}$* GCP-like cells compared to *SmoM2*. **B**. Bar plots showing top 10 upregulated and downregulated Gene ontology (GO) terms (biological processes) in GCP-like cells from *SmoM2-Pten$^{fl/fl}$* mice compared to *SmoM2*. **C**. Bar plots showing top 10 upregulated and downregulated Gene ontology (GO) terms (biological processes) in early GC-like cluster 1 compared to GCP-like cell clusters (3, 4, 8) in *SmoM2-Pten$^{fl/fl}$* tumors. **D**. Bar plots showing the top 10 upregulated and downregulated Gene ontology (GO) terms (biological processes) in macrophages in *SmoM2-Pten$^{fl/fl}$* tumors compared to *SmoM2*. **E.** Violin plots showing expression levels of *Cd36* and *Irf2* in macrophages in the two tumor genotypes.
